# Malaria vector *Anopheles culicifacies* sibling species differentiation using egg morphometry and morphology

**DOI:** 10.1186/s13071-016-1478-5

**Published:** 2016-04-13

**Authors:** Varun Tyagi, A. K. Sharma, Sunil Dhiman, A. R. Srivastava, Ruchi Yadav, D. Sukumaran, O. P. Agrawal, Vijay Veer

**Affiliations:** Vector Management Division, Defence R&D Establishment, Jhansi Road, Gwalior, 474002 Madhya Pradesh India; Medical Entomology Division, Defence Research Laboratory, Tezpur, 784001 Assam India; School of Studies in Zoology, Jiwaji University, Gwalior, 474002 Madhya Pradesh India

**Keywords:** *Anopheles culicifacies*, Malaria, Sibling species, PCR, SEM, Morphometry

## Abstract

**Background:**

The malaria vector *Anopheles culicifacies* (*sensu lato*) is an important malaria vector in Southeast Asia which comprises of five sibling species namely A, B, C, D and E. However, only a few forms have been identified as malaria vectors in various endemic countries. Currently, for the first time egg morphometry and morphology has been used to differentiate the three known vector sibling species of *Anopheles culicifacies* collected from malaria endemic Madhya Pradesh state of central India.

**Methods:**

The adult *An. culicifacies* (*s.l*.) was collected from five districts using standard mosquito collection methods. Adult female mosquitoes were allowed to lay eggs individually. The emerged mosquitoes were identified using allele specific polymerase chain reaction (AS-PCR) to sibling species. Eggs of sibling species A, D and E were studied using scanning electron microscopy (SEM) for morphometric and morphological characteristics.

**Results:**

Currently AS-PCR identified four known sibling species (B, C, D and E) of *An. culicifacies* in the study area. The surface morphology and morphometric attributes of the sibling species A, D and E eggs considerably differed from each other. *An. culicifacies* E had a narrow deck as compared to A and D, while *An. culicifacies* A had a bigger micropyle with 6–7 sectors as compared to D and E that had 6 sectors. *An. culicifacies* D had the smallest float (the structure present on sides of the egg surface in which air is filled that help in floating) and the number of ribs was also fewer than for *An. culicifacies* A and E.

**Conclusions:**

The present study provides the first evidence that in addition to PCR assay, sibling species of *An. culicifacies* can also be differentiated using morphological and morphometric characteristics of the egg stage. The results also advocate that the sibling species of *An. culicifacies* are morphologically dissimilar and can be resolved using advanced microscopy.

## Background

*Anopheles culicifacies* (*sensu lato*) is one of the most important malaria vectors in Southeast Asia and has been recorded in the majority of the malaria affected countries [1, 2]. In India and neighbouring Sri Lanka, *An. culicifacies* (*s.l.*) is considered as a major malaria vector, which contributes significantly high malaria cases annually. It is usually found in rural, semi-urban and tribal settings [3] and prefers breeding in clean water [4–8].

*An. culicifacies* is a complex of five isomorphic species (A, B, C, D and E) [9–14], however, behavioural characters, vectorial capacity, biting preference and susceptibility to malaria parasites of each sibling species is different [10, 14–16]. Since all the sibling species are not vectors, the success of control interventions rely on correct identification of vectors and targeting their breeding sites. Previous studies conducted have demonstrated that identification of sibling species using adult mosquitoes is difficult due to similar morphological characters [17]. Furthermore, identification of sibling species using polytene chromosomes is also not easy as only half-gravid mosquitoes are required for studying the polytene chromosome bandings [11, 17–19]. Molecular methods using PCR assays have been effectively able to identify the sibling species in *An. culicifacies* mosquitoes [2, 17].

As of now the data on egg morphology and morphometry of *An. culicifacies* (*s.l*.) are very limited and not used to identify the species complex. However, the differentiable morphological and morphometric characters occurring in the egg stages of *An. culicifacies* (*s.l*.) can be exploited to differentiate the sibling species. Previous studies have shown that *Anopheles* spp. egg surface morphology and morphometric characteristics can  be useful in separating closely related species. A discriminant function analysis of egg characteristics of the five known species of the *An. quadrimaculatus* Say complex successfully permitted correct classification of 97.7 % of the eggs to species [20]. In other studies, the species complex of various anopheline species including *An. dirus* [21], *An. gambiae* complex [22], *An. maculipennsis* Meigen complex [23, 24], *An. punctimacula* [25], and *An. stephensi* [26] were described using scanning electron microscopy of egg stage. The eggs of *Aedes aegypti* and *Ae. albopictus* [27], *Culex tritaeniorhynchus* and *Cx. quinquefasciatus* [28] have also been used to describe the species.

In the present study, *An. culicifacies* (*s.l*.) were identified to sibling species using AS-PCR assay and thereafter the egg morphology and morphometric characteristics observed using SEM have been used to differentiate *An. culicifacies* species A, D and E. In addition to the strengthening of existing PCR based molecular methods, the present study demonstrates a new microscopy - based approach for *An. culicifacies* sibling species differentiation.

## Methods

### Mosquito collection, rearing and morphological identification

**Fig. 1:**
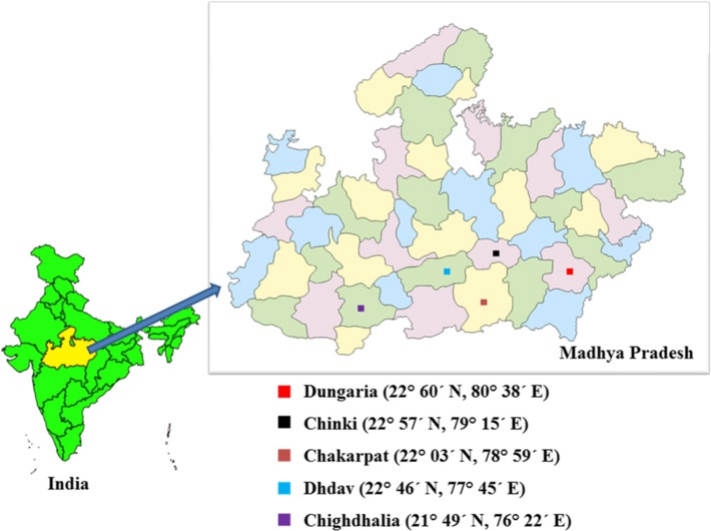
Study area. Map of Madhya Pradesh (India) depicting *An. culicifacies* mosquito collection sites

The adult *An. culicifacies* (*s.l*.) mosquitoes were collected from five malaria endemic districts of Madhya Pradesh (Fig. 1, Table 1). These locations are tribal dominated, situated along the streams of River Narmada and record higher incidence of malaria annually [8, 29–31]. The adult females were collected in the human houses and mixed dwellings (where humans and cattle live together) during 06:00–08:00 h using hand - held aspirators and torch lights. The field - collected adult female *An. culicifacies* (*s.l*.) mosquitoes were brought alive to the mosquito laboratory at The Defence Research Development Establishment, Gwalior (Madhya Pradesh), kept individually in separate cages and allowed to lay eggs. Morphological identification of collected adult mosquitoes was carried out following standard keys [32]

**Table 1:** GPS location of collection sites and details of *An. culicifacies* mosquitoes collected in the study

District/collection site	GPS location	*An. culicifacies* species (N)
Mandla/Dungaria	22° 60′ N, 80° 38′ E	E (54)
Chindwara/Chakarpat	22° 03′ N, 78° 59′ E	E (35)
Hoshangabad/Dhdav	22° 46′ N, 77° 45′ E	C (16), E (68)
Narsinghpur/Chinki	22° 57′ N, 79° 15′ E	D (85)
Khandwa/Chighdhalia	21° 49′ N, 76° 22′ E	D (74)

*N* total number identified

Adult mosquitoes were maintained in the laboratory at 27 ± 1 °C, 75 ± 5 % relative humidity and 12:12 h light: dark period, and provided 10 % sugar solution *ad libitum* through cotton wicks. Rabbits with a shaved belly portion were offered as a blood source to the mosquitoes twice a week. Maintaining of the mosquito culture facility using rabbit, mouse and fowl as blood source has been approved by the institutional committee for animal care (registration number 37/GO/Rbi/S/99/CPCSEA and study protocol number VM-02/51/DS). One portion of the eggs of individual mosquitoes was stored for SEM analysis, while the other portion was kept for the emergence. Of the emerged mosquitoes, at least twenty specimens from each egg batch were used for allele specific polymerase chain reaction (AS-PCR) assay for the identification of *An. culicifacies* sibling species [2, 33, 34]. The field-collected female mosquitoes originally used to lay eggs were identified to *An. culicifacies* species using AS-PCR assay.

For the current study we could not collect *An. culicifacies* species A from the field, therefore, the mosquito colony of *An. culicifacies* species A was obtained from The National Institute of Malaria Research (NIMR), Delhi (India) and maintained in the laboratory for this study after confirmation of identification using AS-PCR assay.

### DNA isolation and PCR assay

For DNA extraction, each adult female mosquito was homogenized in 100 μl lysis buffer (0.1 M Tris–HCl, 0.05 M EDTA, 0.2 M Sucrose, 0.05 % SDS, 0.1 M NaCl). The homogenate was immediately kept on ice for 10 min followed by heat treatment in a water bath at 65 °C for 30 min. Subsequently, 30 μl (5 M) potassium acetate was added and immediately transferred to ice for one hour. The homogenate was centrifuged at 13,000 rpm at 10 °C for 15 min. DNA was precipitated by adding a double volume of ice cooled ethanol and stored at -20 °C overnight. This was then centrifuged again at 13,000 rpm at 10 °C for 15 min and washed in 70 % ethanol. The DNA pellet at the bottom of the eppendorf tube was air-dried, suspended in freshly prepared 50 μl Tris-EDTA (TE) buffer and used as a template for AS-PCR assay.

Initially a multiplex PCR was performed using D3A, D3B, ACA and ACB primers [2, 33, 35]. Primer set D3A and D3B targets D3 domain of 28S subunit of rDNA whereas ACA and ACB primers are allele specific primers specific to *An. culicifacies* species A/D and B/C/E [2, 35]. The PCR amplification was performed in a total reaction volume of 15 μl consisting of Tris–HCl 10 mM (pH 9.0), KCl 50 mM, MgCl_2_ 2 mM, dNTP 0.2 mM, 10 pmoles of each primer, 0.5 unit of Taq polymerase (MBI Fermentas) and 2 μl of genomic DNA. PCR conditions involved an initial denaturation for 5 min at 95 °C followed by 35 cycles of 30 s at 95 °C, 30 s at 55 °C and 60 s at 72 °C sequentially. Thereafter, a final extension step was performed at 72 °C for 7 min. The details of the primers used, type of PCR assay performed and band size obtained are shown in Table 2.

**Table 2:** Details of primers, PCR type performed and band size (bp) obtained for *An. culicifacies* sibling species in the present study

Sequence (5′-3′)	Primer	AS-PCR Type	Band size for different sibling species of *An. culicifacies*
GAC CCG TCT TGA AAC ACG GA	D3A	D3 PCR differentiates sibling species in two groups one is A & D group and second is B, C & E group	382 & 313 bp for species A & D
TCG GAA GGA ACC AGC TAC TA	D3B		385 & 133 bp for species B, C & E
GCC GTC CCC ATA CAC TG	ACA		
CCG TAA TCC CGT GAT AAC TT	ACB		
CTA ATC GAT ATT TAT TAC AC	ADF	A/D-PCR differentiates sibling species A & D individually	359 bp for species A
TTA CTC CTA AAG AAG GC	ADR		248 bp for species B
TTA GAG TTT GAT TCT TAC	DF		248 & 95 bp for species C
AAA TTA TTT GAA CAG TAT TG	BCEF	B/C/E- PCR differentiates sibling species B, C & E individually	359 & 166 bp for species D
TTA TTT ATT GGT AAA ACA AC	BCR		248 & 178 bp for species E
AGG AGT ATT AAT TTC GTC T	CR		
GTA AGA ATC AAA TTC TAA G	ER		

*An. culicifacies* species A and D from the samples which yielded A & D specific bands were differentiated by employing three set of primers (ADF, DF, ADR), whereas four primers, namely CR, ER, BCR, BCEF were used to differentiate the remaining three species B, C and E (which yielded B, C & E specific band) in multiplex AS-PCR assays in the present study (Table 2). For A/D-PCR assay, 37 cycles of initial denaturation at 95 °C for 40 s were performed, while annealing was done at 50 °C for 40 s. The reaction extension was carried out at 68 °C for 45 s followed by final extension at 72 °C for 8 min. PCR reaction was performed by using 20 pmol each of ADF, DF, ADR primers, MgCl_2_ 1.5 mM, 0.5 unit taq polymerase and 2 μl of template DNA in 15 μl of total reaction mixture using thermal cycler (Applied Biosystems, GeneAmp 9700, USA). On the other hand B/C/E-PCR was performed by essentially following the similar reaction conditions, however, the primer concentration of 10 pmol was used for ER primers. The PCR products were resolved in 1.5 % agarose gel electrophoresis using ethidium bromide stain and visualized in UV–VIS gel documentation system (Alpha Innotech, FluorChem, Germany).

### Scanning electron microscopy (SEM) studies

Embryonated (36 h old oviposited) eggs were placed in 2.5 % glutaraldehyde in phosphate buffer (PB) (pH 7.4) at 40 ^o^ C, washed with PB (10 min, with two changes), and fixed in 1 % osmium tetroxide for 1 h at room temperature. The eggs were dehydrated twice by passage through ethanol series as described elsewhere (10 min each for 30 %, 50 %, 70 % and 80 % ethanol, 15 min for 95 % ethanol, 10 min for absolute ethanol) [36, 37]. After dehydration, the dried eggs were mounted on stubs and sputter-coated with gold, and examined under scanning electron microscope (SEM) (FEI – Quanta 400, Netherlands). The terminology and description for the eggs were followed as described previously [38].

### Morphological and morphometric analysis of egg characters

For the morphometric study, 23 attributes of eggs, including length of egg, width of egg, length/width ratio of egg, maximum length of deck, maximum width of anterior deck, maximum width of middle deck, area of deck, area of micropylar disc, area/sector number ratio of micropylar disc, number of sector of micropylar disc, number of antero-ventral tubercles, number of postero-ventral tubercles, number of lobes of antero-ventral tubercles, number of lobes of postero-ventral tubercles maximum length of float, maximum width of float, length/width ratio of egg, number of float ribs, float length as % of egg length, float length /egg width ratio, float width /number of ribs ratio, float length /number of ribs and float width /egg width ratio, were considered.

### Statistical analysis

All morphometric data are expressed as the mean ± SE (standard error of the mean). Variation in the morphometric attributes among *An. culicifacies* species were determined by using one-way ANOVA (analysis of variance) procedure using SigmaStat ver. 3.5.

## Results

### Collection of adult mosquitoes and identification

**Fig. 2:**
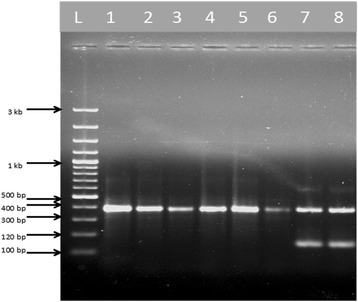
Multiplex D3 PCR for differentiating A, D (382 + 313 bp) and B, C, E (385 + 133 bp) group of *An. culicifacies* sibling species. L: 100 bp ladder, Lanes 1–6: 382 and 313 bp products of species A and D, Lanes 7–8: 385 and 133 bp products of species B, C and E

**Fig. 3:**
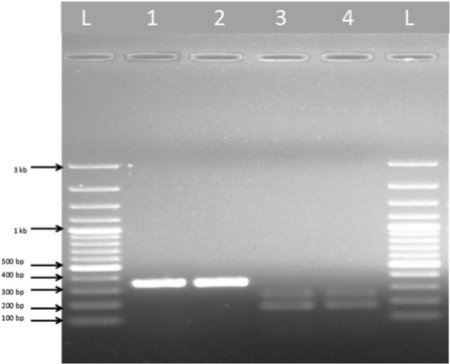
Gel image showing *An. culicifacies* sibling species species A and E. L: 100 bp ladder, Lanes 1–2: 359 bp products of species A, Lanes 3–4: 248 and 178 bp products of species E

**Fig. 4:**
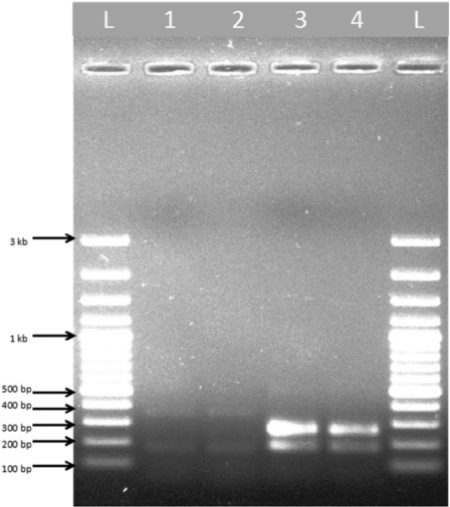
Image showing *An. culicifacies* sibling species D and E. L: 100 bp ladder, Lanes 1–2: 359 and 166 bp products of species D, Lanes 3–4: 248 and 178 bp products of species E

In the present study, a total of 316 adult female *An. culicifacies* (*s.l*.) were collected and identified morphologically. The mosquitoes were used in multiplex PCR assays using allele specific primers for *An. culicifacies* sibling species. The amplification using *An. culicifacies* A/D specific primer ACA with primer D3B produced a 313 base pair amplicon, whereas species B/C/E specific primer ACB along with primer D3A produced a 133 base pair amplification product. The external primer pair D3A and D3B yielded a single product of base pair size 382 for species A and D, and 385 base pair for species B, C and E (Fig. 2). Furthermore, the amplification in A/D-PCR using ADF, ADR and DF primers produced 359 base pair single band for species A, while 166 and 359 base pair bands for species D. In B/C/E-PCR, the amplification produced a 248 base pair single band for species B, 95 and 248 bands for species C, and 178 and 248 base pair size bands for species E respectively (Figs. 3 and 4). The detail of primers used, type of PCR performed and bands obtained for *An. culicifacies* sibling species has been provided in Table 2.

### Morphometry and morphology

For the egg morphology and morphometric study, we used *An. culicifacies* species A, D and E, as the field - collected specimen of species B and C were insufficient and could not be reared in the laboratory despite the efforts. The morphometric values of eggs expressed as the mean ± SE are provided in Table 3, whereas the morphologically differentiable characteristics of the eggs of species A, D and E are presented in Table 4.

**Table 3:** Morphometric attributes of *Anopheles culicifacies* sibling species A, D and E eggs

Abbreviations	*An. culicifacies* A Mean ± SE (Range)	*An. culicifacies* D Mean ± SE (Range)	*An. culicifacies* E Mean ± SE (Range)	*F*-value	*P*-value
Egg attributes					
EGGL	415.75 ± 3.63^a^ (406.00–432.00)	391.24 ± 8.94 (366.30–411.70)	409.03 ± 2.79 (404.80–414.30)	4.532	0.037
EGGW	124.42 ± 2.82^a^ (114.00–134.00)	137.76 ± 3.40 (128.70–145.18)	140.13 ± 6.55 (130.00–152.40)	5.315	0.024
ELWR	3.34 ± 0.06 (3.10–3.56)	2.84 ± 0.07 (2.64–3.10)	2.92 ± 0.12 (2.72–3.14)	13.072	0.001
Deck attributes					
DECL	400 ± 6.06^a^ (380.00–416.00)	340.80 ± 15.95 (316.00–403.92)	395.50 ± 4.37 (390.30–404.20)	8.599	0.007
DECW	41.20 ± 1.96^a^ (36.00–48.00)	44.12 ± 4.74 (40.20–54.00)	15.60 ± 0.74 (14.16–16.64)	21.166	< 0.001
DEMW	33.60 ± 2.78^a^ (28.00–44.00)	34.96 ± 2.79 (29.84–40.00)	9.93 ± 1.00 (8.00–11.40)	23.440	< 0.001
DECA	14,968 ± 1016.04a (12,800.00–18,768.00)	12,824.75 ± 115.48 (10,287.00–14,852.00)	6174.90 ± 349.48 (5526.90–6725.80)	18.409	< 0.001
Ventral tubercles					
ATUN	3.75 ± 0.25 (3.00–5.00)	2.60 ± 0.24 (2.00–3.00)	3.20 ± 0.20 (3.00–4.00)	5.606	0.015
PTUN	3.12 ± 0.12 (3.00–4.00)	2.60 ± 0.24 (2.00–3.00)	2.60 ± 0.40 (2.00–4.00)	1.742	0.209
ATLN	5.62 ± 0.15 (5.00–6.33)	7.24 ± 0.64 (6.00–9.00)	7.10 ± 0.29 (6.66–7.66)	8.036	0.006
PTLN	5.85 ± 0.35 (5.85–8.00)	7.23 ± 0.64 (6.00–9.00)	7.00 ± 0.28 (6.50–7.50)	2.920	0.093
Micropyle					
MICA	326.32 ± 10.41a (291.00–380.00)	252.52 ± 7.21 (236.60–271.60)	226.60 ± 20.05 (188.60–256.70)	19.114	< 0.001
MICDR	50.88 ± 1.10 (46.98–54.35)	42.05 ± 1.19 (39.42–45.26)	37.76 ± 3.34 (31.43–42.78)	17.453	< 0.001
MIDN	6.42 ± 0.20 (6.00–7.00)	6.00 + 0.00 (6.00–6.00)	6.00 + 0.00 (6.00–6.00)	2.062	0.174
Float attributes					
FLOL	283.07 ± 4.27^a^ (264.00–297.00)	250.06 ± 6.73 (232.00–268.94)	293.57 ± 4.22 (285.60–300.00)	15.445	< 0.001
FLOW	73.70 ± 2.04^a^ (70.00–82.92)	66.12 ± 2.48 (59.40–71.40)	89.76 ± 2.74 (84.90–94.40)	18.266	< 0.001
FLWR	3.86 ± 0.11 (3.30–4.22)	3.85 ± 0.11 (3.66–4.06)	3.27 ± 0.13 (3.02–3.47)	5.718	0.020
FRIN	24.0 ± 0.95 (22.00–27.00)	18.60 ± 0.40 (18.00–20.00)	22.00 ± 0.57 (21.00–23.00)	11.833	0.001
FLELP	68.0 ± 1.32 (61.11–71.42)	63.89 ± 0.88 (61.55–65.90)	71.76 ± 1.44 (68.90–73.50)	7.170	0.009
FLEWR	2.25 ± 0.05 (2.09–2.49)	1.82 ± 0.03 (1.74–1.91)	2.10 ± 0.12 (1.87–2.30)	14.710	< 0.001
FWRR	3.09 ± 0.12 (2.66–3.63)	3.62 ± 0.10 (3.30–3.75)	4.08 ± 0.19 (3.69–4.29)	3.436	0.066
FLPR	11.94 ± 0.41 (10.52–13.45)	13.44 ± 0.53 (11.60–14.94)	13.36 ± 0.46 (12.83–14.28)	3.436	0.066
FWEWR	0.58 ± 0.01 (0.53–0.63)	0.47 ± 0.01 (0.43–0.52)	0.64 ± 0.02 (0.61–0.70)	13.898	< 0.001

*EGGL* length of egg, *EGWL* width of egg, *ELW*R length/width ratio of egg, *DECL* maximum length of deck, *DECW* maximum width of anterior deck, DEMW maximum width of middle deck, DECA area of deck, *MICA* area of micropylar disc, *MICDR* area/sector number ratio of micropylar disc, *MIDN* number of sector of micropylar disc, *ATUN* number of antero-ventral tubercles, *PTUN* number of postero-ventral tubercles, *ATLN* number of lobes of antero-ventral tubercles, *PTLN* number of lobes of postero-ventral tubercles *FLOL* maximum length of float, *FLOW* maximum width of float, *FLWR* length/width ratio of egg, *FRIN* number of float ribs, *FLELP* float length as % of egg length, *FLEWR* float length / egg width ratio, FWRR float width / number of ribs ratio, *FLRR* float length / number of ribs, *FWEWR* float width / egg width ratio. ^a^ = μm, a = μm^2^

**Table 4:** Comparative description of eggs of *An. culicifacies* species A, D and E showing morphological differences

	*An. culicifacies* ‘A’	*An. culicifacies* ‘D’	*An. culicifacies* ‘E’
Colour	Black	Black	Black
Shape	Boat-shaped in lateral, ventral and dorsal views	Broadly boat-shaped in both ventral and dorsal views	Broadly boat-shaped in both ventral and dorsal views
Ventral and dorsal surface	Ventral surface concave, dorsal surface curved	Ventral surface concave, dorsal surface strongly curved	Ventral surface slightly concave, dorsal surface curved
Anterior and posterior ends	Anterior and posterior ends blunt, sometimes pointed	Anterior end blunt, posterior end slightly pointed and sometimes blunt	Anterior end blunt, posterior end slightly pointed, sometimes blunt
Anterior lobed ventral tubercles	Usually oval	Usually oval or oblong, but occasionally round	Usually oval or oblong
Posterior lobed tubercles	Similar in structure to anterior lobed tubercles	Similar in structure to anterior lobed tubercles	Similar in structure to anterior lobed tubercles but sometimes found to be round
Number of antero-ventral tubercles	3–7	2–3	3–4
Number of postero-ventral tubercles	1–2 No. less from No. of antero-ventral tubercles	2–3 (Similar in No. to No. of antero-ventral tubercles)	Only 1 No. less from No. of antero-ventral tubercles
Micropyle	Outline of micropylar collar irregular in shape and well developed	Micropylar collar irregular in outline, with smooth surface	Outline of micropylar collar irregular in shape, with slight striations
Number of sectors of micropylar disc	Bigger micropyle with 6–7 sectors	Only 6 sectors	Only 6 sectors
Floats	Short and closer to ventral than dorsal surface	Relatively short and narrow in dorso-ventral plane	Relatively long and wide in dorso-ventral plane
Deck	Continuous, slightly narrows at middle of float, anterior part of deck usually as wide as posterior part	Continuous, anterior part of deck usually wider than posterior part	Continuous and anterior part of deck usually wider than middle part

**Fig. 5:**
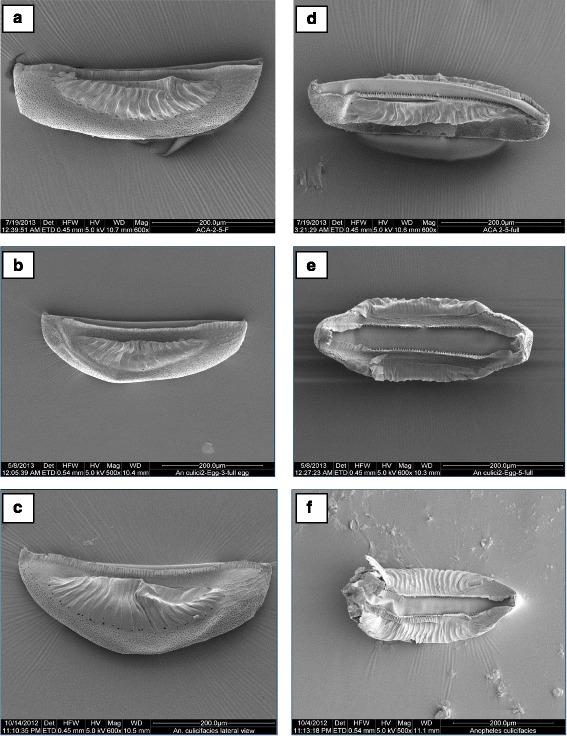
Whole eggs of *An. culicifacies.* **a**, **b**, **c** - lateral aspect showing egg length and float of *An. culicifacies* sibling species A, D and E, respectively; **d**, **e**, **f** - ventral aspect showing deck area of *An. culicifacies* sibling species A, D and E, respectively
  - Ventral surface: Deck is continuous, slightly narrows at middle of float, anterior part of deck usually as wide as posterior part (Fig. 5d). The maximum length of deck was measured as 400 ± 6.06 μm, while the maximum width of anterior deck was calculated as 41.20 ± 1.96 μm. The maximum width of middle deck was measured as 33.60 ± 2.78 μm and the area of deck region was calculated as 14,968 ± 1016.04 μm^2^.
  - Anterior end, micropyle: As shown in Fig. 5a, the anterior and posterior ends were blunt, sometimes pointed. Lobed ventral tubercles were usually oval (Fig. 7a). The number of lobed ventral tubercles at anterior end of deck was 3.75 ± 0.25, while the number of lobes of each anterior ventral tubercles was 5.62 ± 0.15. The micropylar apparatus was located at the apex of dorsal side of the anterior pole. Outline of micropylar collar was irregular in shape and well developed. Inner edge was uniformly and deeply excavated, peaks between excavations tapering to form radial ridges extending about half way across micropylar disc, dividing disc into sector (Fig. 6 a, b). Sibling species A has bigger micropyle with 6–7 sectors and number of sectors of micropylar disc was calculated as 6.42 ± 0.20. Area of micropylar disc was measured as 326.32 ± 10.41 μm^2^. The ratio between area and sector number of micropylar disc was found to be 50.88 ± 1.10.

1. *An. culicifacies* sibling species A:
  - Size: The average length of eggs was 415.75 ± 3.63 μm and the width at the broadest point was calculated as 124.42 ± 2.82 μm. The ratio between the length and width was found to be 3.34 ± 0.06.
  - Overall appearance: In general, the eggs of *An. culicifacies* species A were black in colour and appeared to be boat-shaped in lateral, ventral and dorsal views. Anterior and posterior ends were blunt, but sometimes pointed. Ventral surface was concave, while dorsal surface was curved.
  - Lateral surface: Floats (the structure present on sides of the egg surface in which air is filled that help in floating) were short and closer to ventral than dorsal surface (Fig. 5a). The number of float ribs was found to be 24.0 ± 0.95. The maximum length and maximum width of float was 283.07 ± 4.27 and 73.70 ± 2.04 μm, respectively. Length and width ratio of float was calculated to be 3.86 ± 0.11. The float length as percentage of egg length was found to be 68.0 ± 1.32. The ratio of float length to egg width and number of ribs was 2.25 ± 0.05 and 11.94 ± 0.41, respectively. Similarly, the ratio of float width to egg width and number of ribs was measured as 0.58 ± 0.01 and 3.09 ± 0.12, respectively.

**Fig. 6:**
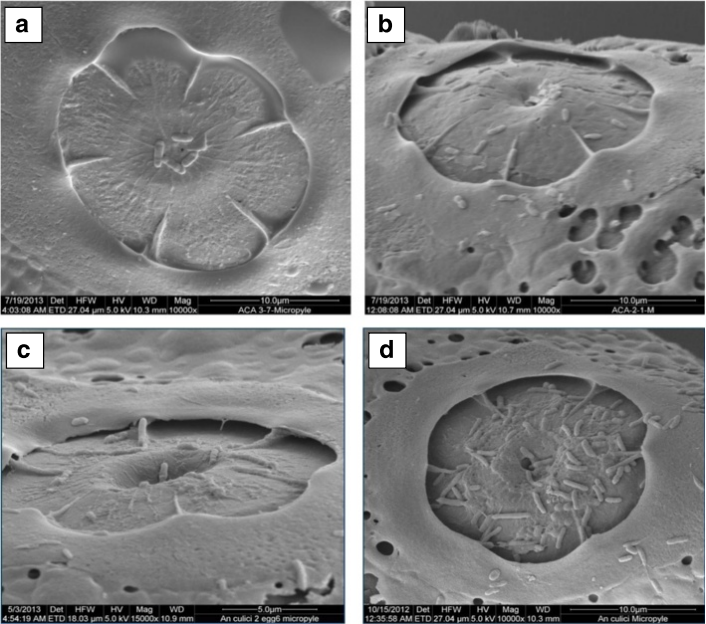
Micropylar disc of *An. culicifacies* sibling species. **a, b:** species A; **c:** species D; **d:** species E
  - Posterior end: The posterior lobed tubercles were similar in structure to anterior lobed tubercles (Fig. 7d). The number of lobed ventral tubercles at the posterior end of deck was 3.12 ± 0.12, while lobes of each posterior ventral tubercle were 5.85 ± 0.35.

**Fig. 7:**
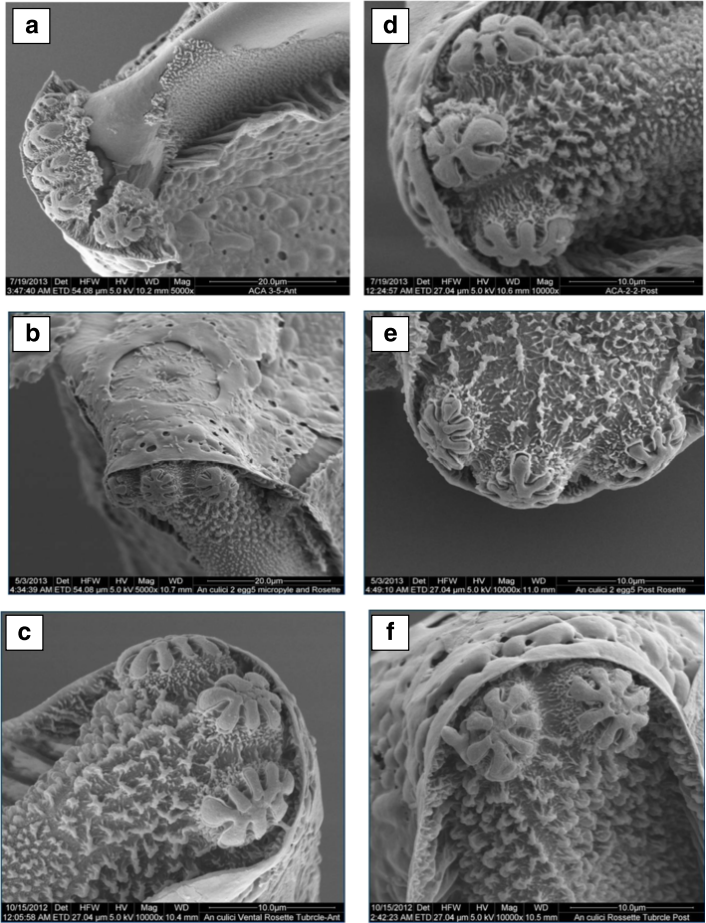
Rosette tubercles. **a**, **b**, **c**: anterior rosette tubercles of *An. culicifacies* sibling species A, D and E, respectively; **d**, **e**, **f**: posterior rosette tubercles of *An. culicifacies* sibling species A, D and E, respectively
2. *An. culicifacies* sibling species D:
  - Size: The mean length of 10 specimens was calculated as 391.24 ± 8.94 μm and width (at the broadest point) as 137.76 ± 3.40 μm. The ratio between length and width was measured as 2.84 ± 0.07.
  - Overall appearance: In general, the eggs of *An. culicifacies* D appeared black in colour, broadly boat-shaped in both ventral and dorsal views. Anterior and posterior ends were slightly pointed, sometimes blunt. Ventral surface was concave and dorsal surface was strongly curved.
  - Lateral surface: Floats were relatively short and narrow in dorso-ventral plane (Fig. 5b), the maximum length of float was measured as 250.06 ± 6.73 μm, and maximum width of float was 66.12 ± 2.48 μm. The number of float ribs was 18.60 ± 0.40. The length width ratio of float was calculated as 3.85 ± 0.11, while float length as percentage of egg length was measured as 63.89 ± 0.88. The ratio between float length and egg width was 1.82 ± 0.03, while the ratio between the float length and number of ribs was 13.44 ± 0.53. The ratio of float width to egg width and number of ribs was calculated as 0.47 ± 0.01 and 3.62 ± 0.10, respectively.
  - Ventral surface: Deck was continuous and the anterior part of deck usually wider than posterior part (Fig. 5e). The maximum length of deck was measured as 340.80 ± 15.95 μm, while maximum width of anterior deck was as 44.12 ± 4.74 μm. The maximum width of middle deck was calculated as 34.96 ± 2.79 μm and the area of deck region was measured as 12,824.75 ± 1115.48 μm^2^.
  - Anterior end, micropyle: Anterior end was blunt, but posterior end slightly pointed and sometimes blunt (Fig. 5b). Lobed ventral tubercles were usually oval or oblong, but occasionally round (Fig. 7b). Number of lobed ventral tubercles at anterior end of deck were 2.60 ± 0.24 and lobes of each anterior ventral tubercle were 7.24 ± 0.64. The micropylar apparatus was located at the apex of dorsal side of the anterior pole and micropylar collar was irregular in outline with smooth surface. The inner edge was uniformly and deeply excavated, peaks between excavations tapering to form radial ridges extending about half way across micropylar disc, dividing disc into sectors (Fig. 6c). In sibling species D, 6 sectors were found. Number of sectors of micropylar disc was 6.00 ± 0.00. Area of micropylar disc was measured as 252.52 ± 7.21 μm^2^. The ratio of area and sector number of micropylar disc was calculated as 42.05 ± 1.19.
  - Posterior end: The posterior lobed tubercles were similar in structure to anterior lobed tubercles (Fig. 7e). Lobed ventral tubercles at posterior end of deck were 2.60 ± 0.24, while lobes of each posterior ventral tubercle were 7.23 ± 0.64.
3. *An. culicifacies* sibling species E:
  - Size: The mean length of eggs of *An. culicifacies* sibling species E was calculated as 409.03 ± 2.79 μm and the width (at the broadest point) as 140.13 ± 6.55 μm. The ratio between length and width was calculated as 2.92 ± 0.12.
  - Overall appearance: In general, the appearance of eggs of *An. culicifacies* E are black in colour and broadly boat-shaped in both ventral and dorsal views, anterior end blunt, posterior end slightly pointed, sometimes blunt. Ventral surface is slightly concave, and dorsal surface is curved.
  - Lateral surface: Floats are relatively long and wide in dorso-ventral plane (Fig. 5c). The maximum length of float was measured as 293.57 ± 4.22 μm, while maximum width of float as 89.76 ± 2.74 μm. The number of float ribs was noted as 22.00 ± 0.57. The length width ratio of float was calculated as 3.27 ± 0.13, while float length as percentage of egg length was calculated as 71.76 ± 1.44. The ratio of float length to egg width and number of ribs was calculated as 2.10 ± 0.12 and 13.36 ± 0.46 respectively. Whereas, the ratio of float width and egg width was calculated as 0.64 ± 0.02, and the ratio between float width and number of ribs was recorded as 4.08 ± 0.19.
  - Ventral surface: Deck was continuous, and the anterior part of the deck was usually wider than the middle part (Fig. 5f). The maximum length of the deck was measured as 395.50 ± 4.37 μm and maximum width of anterior deck was measured as 15.60 ± 0.74 μm, while maximum width of middle deck was calculated as 9.93 ± 1.00. The area of deck region was measured as 6174.90 ± 349.48 μm^2^.
  - Anterior end, micropyle: Anterior end was blunt while posterior end slightly pointed, sometimes blunt (Fig. 5c). Lobed ventral tubercles were usually oval or oblong (Fig. 7c). Lobed ventral tubercles at anterior end of deck were 3.20 ± 0.20, while lobes of each anterior ventral tubercle were 7.10 ± 0.29. The micropylar apparatus was located at the apex of dorsal side of the anterior pole. Outline of micropylar collar was irregular in shape with slight striations, inner edge was deeply but uniformly excavated. Peaks between excavations were tapering to form radial ridges extending about half way across the micropylar disc and dividing disc into sectors (Fig. 6d). In sibling species E of *An. culicifacies*, 6 (6.00 ± 0.00) sectors were recorded in the micropylar disc. Area of micropylar disc was calculated as 226.60 ± 20.05 μm^2^. The ratio of area and sector number of micropylar disc was found to be 37.76 ± 3.34.
  - Posterior end: The posterior lobed tubercles were similar in structure to anterior lobed tubercles but sometimes found to be round (Fig. 7f). Lobed ventral tubercles counted at posterior end of deck were 2.60 ± 0.40, while the number of lobed posterior ventral tubercles counted was found to be 7.00 ± 0.28.

## Discussion

Differences in biological characteristics of different members of the sibling species complexes have significant bearing on the malaria transmission dynamics. In all 23 taxa of anopheline sibling species have been recognized across the world bearing distinct gene pools and hence differing in biological characteristics that determine their disease transmission potential. All the sibling species are not capable of transmitting malaria, therefore differentiating sibling species using correct markers is very important for planning effective control strategies and understanding of their role in disease transmission.

Previous studies have attempted to find out the morphological markers for the members of *An. culicifacies* species complex and reported variation in the spermatheca of species A and B [39], but could not successfully differentiate all the sibling species. Cytogenetic methods involving polytene chromosome and mitotic karyotyping have been extensively used to differentiate existing five sibling species of *An. culicifacies*. However, both methods have some shortcomings that may result in inaccurate identification of sibling species. Studies have evidenced that *An. culicifacies* species E cannot be differentiated from species B as they comprise of homosequential polytene chromosome arrangements [14]. Molecular methods based techniques using rDNA and COII such as AS-PCR, and gene-specific PCR-RFLP have been extensively used in differentiation and identification of sibling species [2, 33, 35]. These methods seem to be accurate but suffer certain disadvantages such as high running cost, requirement of different sophisticated instruments, and specially trained manpower, making them practically difficult in routine identification.

During recent years, high resolution scanning electron microscopy (SEM) describing various developmental stages and specific organs of insects have been used comprehensively, as it offers a more precise and convincing description useful to illustrate a species using a realistic approach [40–44]. The surface morphology and morphometric characteristics of mosquito eggs have significance in the study of identification and differentiation of various sibling species and received very little attention until Hinton [45] recognized the potential of SEM for visualizing egg microstructures to emphasize and describe morphological characteristics for species recognition [46]. Many species including anopheline [20, 22, 36, 47, 48], culicine [28, 37] and aedini mosquitoes [49] have been studied for their identification and differentiation based on egg morphology and morphometrics using SEM. Similarly, SEM studies of *Anopheles* spp. eggs have been useful for differentiating sibling species [21] as well studying the relationship among species groups [46, 50] and complexes [20].

In the present study, we have employed a simple and reproducible two step multiplex AS-PCR-based assay to differentiate four (B, C, D, E) field-collected and laboratory reared species A of *An. culicifacies*. The assay was able to successfully identify all the species indicating the robustness of the method and implying that the assay could be used undoubtly to differentiate the different members of the *An. culicifacies* species complex in various geographical areas. However, the aim of the current study was not limited only to optimize a reproducible and simple PCR method to distinguish sibling species, but also to discover the differentiable morphological and morphometric characteristics of the eggs that could be exploited to identify the species within the complex. Therefore, considering the AS-PCR assay as a standard method, the microscopic characteristics of a known malaria vector, *An. culicifacies* species were compared to identify confirmatory characteristics for sibling species. SEM analysis of attributes on the surface morphology and morphometry of the eggs of *An. culicifacies* sibling species A, D and E showed that all of them were significantly different from each other based on egg dimensions, ventral tubercles, micropyle along with the deck and float attributes. Results suggested that species E has narrow deck as compared to species A and D. In Africa, studies on *An. gambiae* complex showed that width of the deck region had been successfully used for many years to distinguish eggs of *An. melas* from those of fresh water *An. gambiae* (*s.l*.) [51–53], although some freshwater *An. gambiae* from inland Nigeria produced eggs with melas-like morphology [54]. Species D has a small float and the ribs are also fewer than in species A and E, similar to the observations made previously [45] in the eggs of *An. gambiae* complex in which it was noted that saltwater forms generally have smaller eggs with proportionally smaller floats and fewer ribs than freshwater counterparts. Different sibling species of *An. dirus* have shown that the float of species B is slightly larger than that of species A, although this structure has well defined ridges in both species, and also the egg of species C bears floats with markedly pointed ends whereas the float of species D is shorter than those of the other species [21]. Hinton [45] also gave the quantification, which indicated a difference in the number of anterior versus posterior lobed tubercles for *An. gambiae* (6 *vs* 5), but not for *An. merus* (5 *vs* 5). Similar to the observation made for *An. gambiae,* [45] the present study also found a general trend of more anterior than posterior lobed tubercles among the *An. culicifacies* sibling species A, D and E. Apart from this, it was also observed that the shape and number of lobes of both anterior and posterior tubercles also vary among themselves. Among egg attributes, the micropylar apparatus has been found to be a prominent feature for species confirmation in several anopheline species [22, 25] and culicines [28]. Currently we have observed that *An. culicifacies* sibling species A has equal or more micropyle sectors (6 and/or 7) as compared to species D (6) and species E (6). Hinton [45] also reported similar findings of different numbers of micropylar sectors in *An. gambiae* eggs showing more micropylar sectors/ ridges (7) than *An. nzelas* (6).

The present study is the first attempt to use egg characteristics to differentiate the sibling species of *An. culicifacies* and in addition to optimizing and validating a robust AS-PCR assay, the study also describes various prominent egg characters useful for differentiating the three sibling species. The present study had the limitation that it could not include all the sibling species of *An. culicifacies*; however, it adds important information to existing knowledge on the identification of *An. culicifacies* sibling species complex and could be useful effectively in designing malaria control programmes in the regions where *An. culicifacies* has been regarded as a major vector of malaria.

## Conclusions

The AS-PCR based assay employed currently was sensitive and highly useful in identifying and differentiating all the sibling species of *An. culicifacies* in the study area. Morphology and morphometry of eggs of *An. culicifacies* sibling species established that the sibling species differ considerably from each other. The information generated in this study would be useful in identifying the *An. culicifacies* sibling species not only in India but also elsewhere. The different egg characteristics identified can be used as stand alone criteria for identifying the *An. culicifacies* sibling species complex to devise effective control against this potential malaria vector.
